# Predictive Value of Gene Polymorphisms on Recurrence after the Withdrawal of Antithyroid Drugs in Patients with Graves’ Disease

**DOI:** 10.3389/fendo.2017.00258

**Published:** 2017-09-29

**Authors:** Jia Liu, Jing Fu, Yan Duan, Guang Wang

**Affiliations:** ^1^Department of Endocrinology; Beijing Chao-Yang Hospital, Capital Medical University, Beijing, China

**Keywords:** gene polymorphism, Graves’ disease, antithyroid drugs, recurrence, hyperthyroidism

## Abstract

Graves’ disease (GD) is one of the most common endocrine diseases. Antithyroid drugs (ATDs) treatment is frequently used as the first-choice therapy for GD patients in most countries due to the superiority in safety and tolerance. However, GD patients treated with ATD have a relatively high recurrence rate after drug withdrawal, which is a main limitation for ATD treatment. It is of great importance to identify some predictors of the higher recurrence risk for GD patients, which may facilitate an appropriate therapeutic approach for a given patient at the time of GD diagnosis. The genetic factor was widely believed to be an important pathogenesis for GD. Increasing studies were conducted to investigate the relationship between gene polymorphisms and the recurrence risk in GD patients. In this article, we updated the current literatures to highlight the predictive value of gene polymorphisms on recurrence risk in GD patients after ATD withdrawal. Some gene polymorphisms, such as *CTLA4* rs231775, human leukocyte antigen polymorphisms (DRB1*03, DQA1*05, and DQB1*02) might be associated with the high recurrence risk in GD patients. Further prospective studies on patients of different ethnicities, especially studies with large sample sizes, and long-term follow-up, should be conducted to confirm the predictive roles of gene polymorphism.

## Introduction

Hyperthyroidism is a common endocrine disease caused by increased synthesis and secretion of thyroid hormone (1). Graves’ disease (GD) is an autoimmune thyroid disease that results from excessive stimulation of the thyroid by circulating TSH receptor antibodies (TRAb), which is the most common cause of hyperthyroidism (1). As antithyroid drugs (ATDs) treatment avoids radiation exposure and has a low risk of hypothyroidism, it is frequently considered as the first-choice therapy for GD patients (2). However, GD patients treated with ATD have a relatively high recurrence rate after drug withdrawal (3, 4). The persistent or recurrent hyperthyroidism results in increased medical expenses and a wide spectrum of complications, such as atrial fibrillation, heart failure, and osteoporosis, even a long-term and negative impact on the quality of life (5, 6). Thus, it is of great importance to identify some predictors, prior to ATD treatment, which can indicate a higher risk of recurrence. If the likelihood of recurrence after ATD treatment is high, radioiodine therapy or thyroidectomy might be more preferable. This strategy would facilitate an appropriate therapeutic approach for a given patient at the time of GD diagnosis.

The genetic factor was widely believed to be an important pathogenesis for GD (7). Increasing studies were conducted to investigate the relationship between gene polymorphisms and the recurrence risk in GD patients after ATD withdrawal (8–13). In this article, we updated the current literatures to highlight the predictive value of gene polymorphisms on recurrence risk in GD patients after ATD withdrawal.

## The High Recurrence Risk in GD Patients after ATD Withdrawal

There are three treatment options for GD patients, including ATD, radioiodine therapy, and thyroidectomy (1). ATD treatment is frequently used as the first-choice therapy for GD patients in most countries (2). However, the recurrence rate of ATD treatment is approximately 50–60%, which is relatively higher as compared with radioiodine therapy or thyroidectomy (3, 4). The previous studies have demonstrated that the recurrence risk varies in GD patients with different clinical characteristics (4, 9, 14, 15). Therefore, the risk predictors for recurrence should be taken into account when choosing an appropriate therapeutic approach for every GD patient. Previous studies have shown that young age, high concentrations of TRAb, large goiter size, and severe thyrotoxicosis were associated with higher recurrence risk (4, 9, 14, 15). Unfortunately, the predicting ability of these factors is unsatisfactory.

## The Relationship Between Gene Polymorphisms and Recurrence Risk of GD Patients

Several studies have found that the occurrence of GD appeared in family aggregation (16). The evidence from further genetic studies showed that genetic factors are related to the pathogenesis of GD (7). Some immune-regulatory genes, including human leukocyte antigen (HLA), *CD40*, cytotoxic T-lymphocyte-associated factor 4 (*CTLA4*), protein tyrosine phosphatase, non-receptor type 22 (*PTPN22*), and Fc receptor-like protein 3 (*FCRL3*), have been observed to be involved in the development of GD (7). In addition, thyroid autoantigen genes are also related to the development of GD (7). Thus, researchers want to find some genetic predictors for the recurrence risk in GD patients after ATD withdrawal.

Recently, the relationship between gene polymorphisms and the recurrence risk in GD patients after ATD withdrawal have been investigated (8–13). We carried out a computerized literature search in PubMed, EMBASE, Web of Science, Cochrane database, and reference lists of relevant studies up to July 20, 2017. The keywords of retrieval were (“polymorphism” or “variant” or “mutation” or “gene” or “genotype”) and (“antithyroid drug” or “antithyroid medicine” or “antithyroid agent” or “methimazole” or “propylthiouracil” or “carbimazole”), in combination with (“hyperthyroidism” or “Graves’ disease” or “thyrotoxicosis”), without language or region restriction. In general, the ATD treatment duration is recommended as 12–18 months, so the recurrence of GD was defined as the recurrence of hyperthyroidism after at least 12 months of ATD treatment (17). The eligible studies were observational cohort or case-control studies and were identified according to the following criteria: (1) studies that investigated the association between gene polymorphism and the recurrence in GD patients after ATD withdrawal; (2) the duration of ATD therapy was 12 months or more; (3) TRAb was negative when stopping ATD treatment; (4) the follow-up duration was at least 12 months after ATD withdrawal. The exclusion criteria included: (1) duplicated studies; (2) case, reviews, letter, or books; and (3) unavailable data.

**Figure 1:**
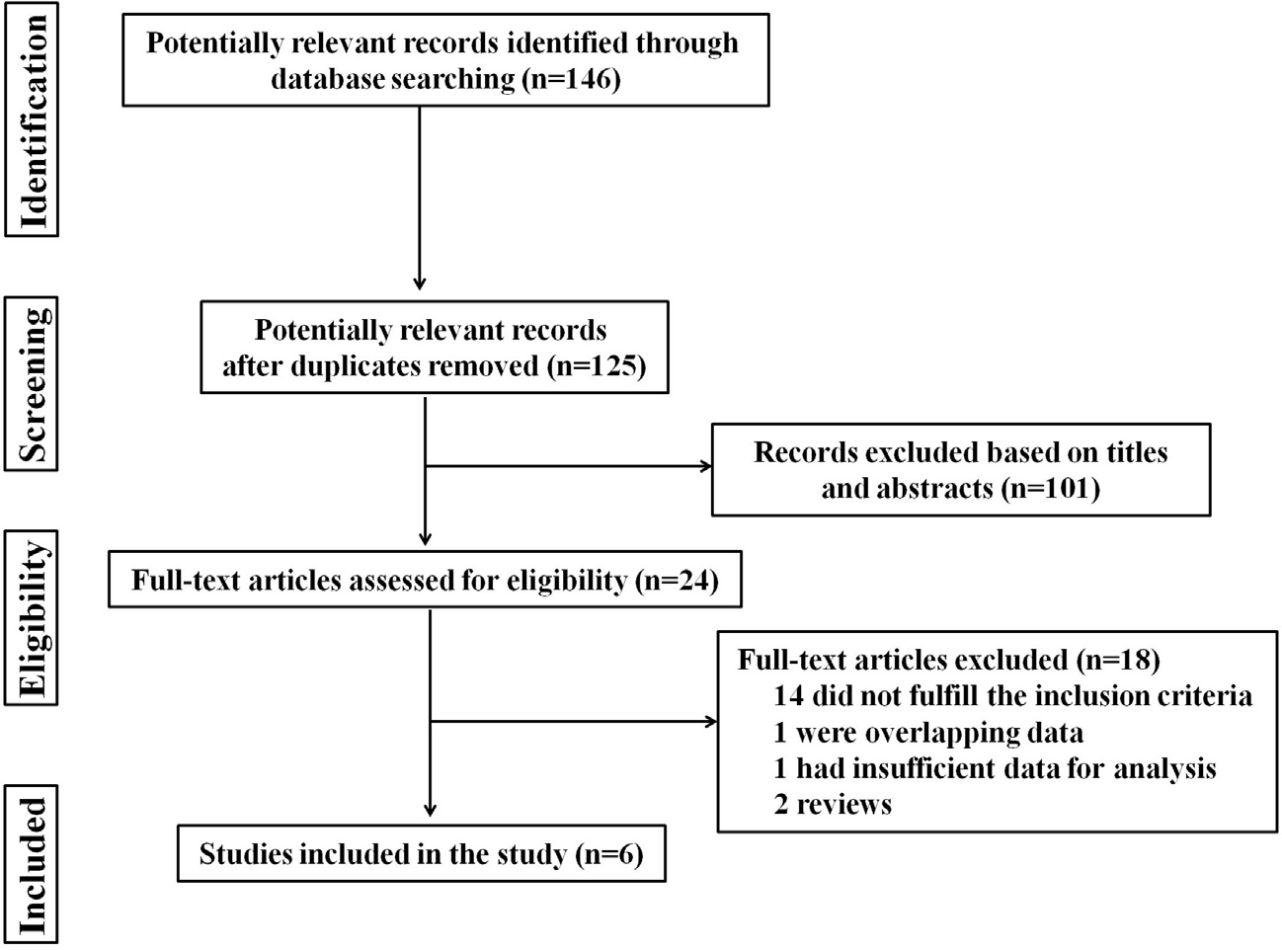
Flow chart of literature search and study selection.

A total of 146 studies were identified through database searching. However, after manually screening the titles and abstracts, 24 studies were chosen after excluding duplicates and irrelevant papers. The remaining 24 studies were reviewed in detail. Fourteen of these were removed because they did not fulfill the inclusion criteria, and four were excluded due to overlapping data, insufficient data for analysis, or other reasons. The flowchart, including study identification, inclusion, and exclusion factors is shown in Figure 1. Finally, only 6 studies, including 4 observational cohort studies and 2 case-control studies, with a total of 1,398 GD patients, were included (8–13).

The main characteristics of the included studies are shown in Table 1. Of the six studies, three were conducted in Asian populations, and three in Caucasian populations. The types of gene polymorphism included: *CTLA4* polymorphisms (rs231775, rs3087243, rs5742909, rs231777, and rs231779), *PTPN22* polymorphism (rs2476601), HLA polymorphisms (DRB1*03, DQA1*05, and DQB1*02), *CD40* polymorphisms (rs745307, rs11569309, rs3765457, rs1883832, rs1535045, and rs4810485), *CD28* polymorphisms (rs1879877 and rs3181113), T393C SNP of Galphas gene (*GNAS1*), and E33SNP of thyroglobulin (*Tg*) (*Tg* E33SNP). The follow-up duration was 12–36 months after withdrawing therapy. Among these studies, two evaluated *CTLA4* rs231775 (8, 9), two evaluated *CTLA4* rs3087243 (8, 9), two evaluated HLA DQA1*05 (8, 13). Only one study evaluated the rest of the gene polymorphisms. Among these gene polymorphisms, *CTLA4* rs231775, HLA polymorphisms (DRB1*03, DQA1*05, and DQB1*02), *CD40* polymorphisms (rs745307, rs11569309, and rs3765457), *PTPN22* polymorphism (rs2476601), T393C SNP of *GNAS1* and *Tg* E33SNP were observed to be associated with the high-recurrence risk in GD patients after ATD withdrawal (8–13).

**Table 1:** Characteristics of included studies in the meta-analysis.

Reference	Year	Ethnicity	Study type	Total patients	Relapse patients	Treatment duration of ATD	Follow-up duration (years)	Genotyping method	The types of gene polymorphism
Vos et al. (8)	2016	Caucasian	Observational cohort study	178	66	1 year	2	PCR-RFLP	*CTLA4* (rs231775, rs3087243); *PTPN22* (rs2476601); HLA (DRB1*03, DQA1*05, DQB1*02)
Wang et al. (9)	2012	Asian	Observational cohort study	262	156	at least 1 year	3	PCR-RFLP/TaqMan	*CTLA4* (rs231775, rs3087243, rs5742909); *CD40* (rs745307, rs11569309, rs3765457, rs1883832, rs1535045, rs4810485); *CD28* (rs1879877, rs3181113)
Glowacka et al. (10)	2009	Caucasian	Case-control study	276	213	1 year	2	PCR-RFLP	T393C SNP of *GNAS1*
Wang et al. (11)	2007	Asian	Observational cohort study	208	120	1–3 years	3	PCR-RFLP	*CTLA4* (rs231777, rs231779)
Hsiao et al. (12)	2007	Asian	Observational cohort study	215	149	1–3 years	3	PCR-RFLP	*Tg* E33SNP
Badenhoop et al. (13)	1996	Caucasian	Case-control study	259	117	1 year	1	PCR-RFLP	HLA DQA1*05

*PCR-RFLP, polymerase chain reaction-restriction fragment length polymorphism; CTLA4, cytotoxic T-lymphocyte antigen 4; PTPN22, protein tyrosine phosphatase, non-receptor type 22; HLA, human leukocyte antigen; GNAS1, Galphas; Tg, thyroglobulin; ATD, antithyroid drug*.

### CTLA4

T-cell abnormality is associated with the pathogenesis of GD (1). The CTLA4 is a main negative-regulatory factor of T-cell-mediated immune responses (18, 19). CTLA4 inhibited T-cell activation and mediated antigen-specific apoptosis of T cells (19). Several studies have revealed that *CTLA4* polymorphisms including rs231775, rs231779, and rs3087243 were likely the susceptibility variants for GD (20–22). A recent observational cohort study showed that there is no association between recurrence risk and the *CTLA4* polymorphism (rs231775, rs3087243) in Caucasians patients with GD (8). However, Wang et al.’ studies showed that *CTLA4* rs231775 was significantly associated with the recurrence of hyperthyroidism in Asians patients with GD (9). On the one hand, the inconsistent results for the association between *CTLA4* polymorphisms and the recurrence in GD patients might be related to the ethnic differences. On the other hand, these two studies were small-scale, which probably also contributed to the divergent results. Then, the follow-up duration was different in the included studies. The recurrence cases will increase if the follow-up duration extends, which might further influence the association between gene polymorphism and the recurrence risk in GD patients.

### Human Leukocyte Antigen

Human leukocyte antigen region contains many immune response genes, which linked HLA to some autoimmune diseases, such as GD. Several studies have demonstrated that HLA DRB1*03, DQB1*02, and DQA1*05 displayed strong associations with GD in Caucasians (23, 24). Recently, some Asian studies also showed the association between HLA polymorphism (DPB1*05:01) and the susceptibility of GD (25, 26). A recent study from Caucasians showed that HLA DRB1*03, DQA1*05, and DQB1*02 polymorphisms were significantly related to the high recurrence risk after ATD treatment in GD patients (8). However, another Caucasians study did not observe the association between recurrence risk and HLA DQA1*05 (13). So far, the study regarding the association between recurrence risk of GD and HLA polymorphism is still absent in Asians populations. The inconsistent results in Caucasians might be involved in the small sizes of included studies and different duration of follow-up.

### CD40

CD40 is mainly expressed on antigen-presenting cells and B cells, as well as on other types of cells, such as thyroid follicular cells (27). CD40 not only interacts with the CD40 ligand (CD40L) on the T cells but also promotes B-cell proliferation and antibody secretion (27, 28). In spite of some inconsistent results, a meta-analysis still confirmed the association between *CD40* polymorphism (rs1883832) and GD (29). So far, only a recent study evaluated the association between *CD40* polymorphism and recurrence risk of GD and showed that *CD40* polymorphisms (rs745307, rs11569309, and rs3765457) were associated with the high recurrence risk of GD patients (9).

### PTPN22

PTPN22 is related to the activity of lymphoid tyrosine phosphatase, which a negative regulator of T-cell activation (30). The association between *PTPN22* polymorphism (rs2476601) and GD has been shown in many studies among Caucasians (30, 31). And a recent Caucasians study has shown that this polymorphism is associated with recurrence risk in GD patients as well (8). Interestingly, *PTPN22* polymorphism (rs2476601) occurs very rarely in Asians and Africans, and no association between *PTPN22* polymorphism (rs2476601) and the onset or recurrence risk of GD was observed in Asians and Africans (32).

### GNAS1

*GNAS1* is a gene that encodes α-subunit of G proteins (33). The T393C SNP of *GNAS1* has been significantly associated with the clinical course in a variety of cancers (33). Interestingly, although not directly involved in the development of GD, T393C SNP of *GNAS1* was shown to be related to the relapse of hyperthyroidism in GD patients after ATD withdrawal (10). The TT genotype of T393C SNP of *GNAS1* was considered to be associated with the increased expression of Gαs mRNA (33). TSH receptor belongs to the G-protein coupled receptor superfamily (1). So, T393C SNP of *GNAS1* might be related to the relapse after ATD withdrawal in GD patients by modulating TSH receptor.

### Thyroglobulin

Thyroglobulin is a main autoantigen of autoimmune thyroid diseases, including both GD and Hashimoto’s thyroiditis (34). Some studies in patients with autoimmune thyroid disease have shown that anti-Tg antibodies are specific toward a restricted number of epitopes on Tg, thus, Tg is important in the pathogenesis of GD due to its specific features (35). In several studies, *Tg* gene polymorphism in exon 33 (*Tg* E33SNP) was related to the increased susceptibility for GD (35, 36). And a recent study also showed the association between *Tg* E33SNP and the recurrence risk of GD (12).

Based on a limited number of studies, *CTLA4* polymorphism (rs231775), HLA polymorphisms (DRB1*03, DQA1*05, and DQB1*02), *CD40* polymorphisms (rs745307, rs11569309, and rs3765457), *PTPN22* polymorphism (rs2476601), T393C SNP of *GNAS1*, and *Tg* E33SNP might be associated with the high recurrence risk in GD patients after ATD withdrawal (8–13). However, there is still some query about the predictive value of gene polymorphisms for the high recurrence risk in GD patients. First, the studies evaluating the association between gene polymorphisms and the recurrence in GD patients after ATD withdrawal were relatively small. Each gene polymorphism was investigated only by one or two studies. Furthermore, most of the included studies were small scale. Then, the quality of the included studies was relatively poor. Further prospective studies with large sample sizes, and long-term follow-up, should be conducted to confirm the predictive roles of gene polymorphism. Despite these conflicting results, one implication is that gene polymorphisms are supposed to an important risk predictor for the recurrence risk in GD patients. In addition, it is worth noting that the etiology of GD is related to genetic and environmental factors, so it is difficult for any single factor to well predict the recurrence risk for a given patients. Thus, a prediction model based on both genetic and environmental risk factors for GD recurrence might better predict the recurrence risk for a given patient.

## Conclusion

Antithyroid drug is a common choice for a new diagnosed GD patient, and some genetic factors may greatly influence therapeutic outcome for GD patients. Based on a limited number of studies, *CTLA4* polymorphism (rs231775), HLA polymorphisms (DRB1*03, DQA1*05, and DQB1*02), *CD40* polymorphisms (rs745307, rs11569309, and rs3765457), *PTPN22* polymorphism (rs2476601), T393C SNP of *GNAS1* and *Tg* E33SNP might be associated with the high recurrence risk in GD patients after ATD withdrawal. Further, prospective studies on patients of different ethnicities, especially studies with large sample sizes, and long-term follow-up, should be conducted to confirm the predictive roles of gene polymorphism.

## Author Contributions

JL and GW conceived and designed the review; JL, JF, YD, and GW performed the review; JL, JF, and YD analyzed the data; JL and GW wrote the paper.

## Conflict of Interest Statement

The authors declare that the research was conducted in the absence of any commercial or financial relationships that could be construed as a potential conflict of interest.
